# Croup and Diphtheria

**Published:** 1860-05

**Authors:** Orren Smith

**Affiliations:** Chicago


### THE CHICAGO MEDICAL EXAMINER

Vol. I. MAY, 1860. No. 5

### ORIGINAL COMMUNICATIONS.

### CROUP AND DIPHTHERIA.

By ORREN SMITH, M. D., of Chicago.

Croup is usually defined to be an inflammation of the mucous membrane, lining the trachea. But it is not desirable or practical to make this close anatomical limitation to the location of this disease. As the inflammation, when commencing in this location, may extend upward to the lining membrane of the larynx, glottis, epiglottis, and the whole membrane lining the throat and fauces; and downward to the membrane lining the bronchia, to its minute divisions, and even of the air cell. And the inflammation may not stop here, but extend to the substance of the lung itself, or to the submucous tissue of the parts about the trachea and throat; making complications which are of importance to notice in the diagnosis and treatment; as they compass inflammations of the mucous, parenchymatous, and cellular tissue, with the modifications of symptoms, and lesions consequent upon the different localities and the laws of inflammation, when affecting such different structures. Nor is it necessary that this inflammation should always commence in the lining membrane of the trachea, for it may commence in the larynx or throat, and extend down ward to the locality of croup, or it may commence below and extend upward to the same, and still give all the phenomena of croup with its different stages.

Croup, when not complicated with lesions of structures above named, is simply an inflammation of the mucous membrane of particular *grades*, as I will attempt to show, and has nothing in its phenomena to authorize its being called *specific* in any acceptation of that term.

It is true, that when croup destroys life, it does so by producing asphyxia, and its consequent congestions and lesions; and these congestions and lesions are in no respect different from those which follow asphyxia from drowning or hanging, except they are more slowly produced, and the consequent congestions may be more strongly marked. It is very seldom, if ever, that the constitutional effects of the inflammation destroy life in croup. Therefore, it is the interference with the function of respiration which we fear, and should guard against. The same amount of inflammation may exist in the mucous membrane of the nasal passages, and be comparatively harmless, even when the membrane lining such passage is so much swollen as to perfectly close the passage, and respiration is performed through the mouth; and still we feel safe, because the blood may still be properly changed; and the constitutional effects are not sufficient to produce fatal lesions. But previously to the age of puberty, and this is almost always the age for croup, the parts about the trachea and larynx are not developed to that degree that they can bear to have the calibre of the trachea closed to any considerable extent without endangering life; and this is an age also when all inflammations are active and rapid in their phenomena and terminations. So that the rapidity with which this disease produces changes, is no evidence of any specific character. And the fatal character of the disease depends more upon this want of development, and the rapid pathological changes incident to the age of the patient, than to any thing peculiar in the phenomena of the disease itself. And it may be further added, that this disease observes all the phenomena which belong to inflammations in these structures, and none others.

And it is the different *stages* of common inflammation, and its complications with the several structures above named, that we should study to diagnose and understand, if we would treat croup successfully. And this leads me to inquire what are the stages of inflammation found in croup? I answer:—

First. The lowest grade of inflammatory excitement found in mucous membranes, one in which there is a want of the ordinary mucous secretion of the membrane involved, and being located about the chordæ vocales and organs of voice, in addition to dryness of the membrane, causes a spasmodic contraction of those parts, and imitates by paroxysms the true croup respiration. And in this stage some slight circumstance, like a change of the temperature of the atmosphere, or a drink of cold water, or warm tea, may so modify the action of the mucous membrane involved, as to restore secretions of a normal character, and carry the pathological action back to a physiological one; and with restoration of function, the spasmodic character may cease entirely, and the ordinary phenomena of catarrhal secretion completely restore normal conditions. This then would be known as spasmodic croup, and depends upon *both a low* grade of inflammation, and a location over parts liable to muscular spasmodic action. For if the grade of inflammation was high, the muscular tissue beneath would be to a greater or less extent involved, and we all know that muscular fibre when involved in inflammation, not only looses its power of spasmodic action, but the ordinary force of common contraction also. But when this low grade of inflammation is located about the larynx, we get this spasmodic warning early in the inflammation, and at a time when some slight indication, as before intimated, may restore the healthy condition. But it is, nevertheless, inflammation, and the spasm only a consequence, a symptom.

But if the early inflammation is located in the trachea, below the larynx, it may pass along unnoticed until the general symptoms of inflammation demand notice, or the parts become so changed by the force of inflammatory action and intensity of disease, as to make the second grade of inflammatory action, or true croup, as it has been called by writers.

This, instead of being a disease of trifling importance, becomes one of grave character, and demands for successful treatment clear and distinct ideas of its pathological character and therapeutic indications, and a knowledge or ability to distinguish when those indications are answered.

In this second grade of croup the inflammation is more intense, probably lower in the trachea, and of longer duration when we are first called to see the case, and the parts involved more changed by pathological action than we would find in the first grade of croup. Here the pulse becomes accelerated and hard, and this hardness of the pulse in all the early stages of croup, before congestions occur from the want of decarbonization of the blood, becomes one of the best criteria to judge of the case and its progress; for so long as the pulse continues *hard*, we have no positive evidence of the safety of the patient, even although the croup may for a time loose its peculiar croup character. When this grade of inflammation has continued a length of time, a secretion containing more albumen or fibrin than pertains to ordinary mucous, is thrown out of the vessels of the inflamed membrane; and is simply the result of the ordinary processes of inflammatory action, is common and analagous in all inflammatory actions of mucous membranes, whether in the nasal passages, trachea, stomach, bowels, or vagina. And we have its analogue in all those inflammations called phlegmonous in the cellular tissue, where something like this same material is thrown from the blood around the parts involved in the inflammation; also in acute rhuematic inflammation, and sub-acute or chronic; also in the earlier stages before the exudation becomes organized. In short, it is the effort of the system for repair, and the only fault with nature is, that the material for repair is thrown into a dangerous locality, because space for it can not be tolerated; hence, in cases where the inflammation is not severe enough to cause a sufficient amount of deposit to be thrown out to fatally block the trachea, the processes of nature are sometimes sufficient for a cure; and this is more common in children who are sufficiently advanced towards puberty to have some increased development of the trachea.

This second character of croup often creeps insiduously upon the patient, and is overlooked, or not appreciated, until the disease has made much progress; for the reason, as before stated, that it is wanting in spasmodic character, and beside, it often has an apparent remission in the morning and forepart of the day, and sometimes leads friends to think that the severity of the disease is passed when such is not the fact. The peculiar croup membrane, or diphtheric deposit, is dense in proportion to the severity of the inflammation up to a certain point of inflammation, beyond which we have another character of secretion and exudation, as we shall have occasion to notice. When this deposit is first thrown upon the inflamed membrane, it may have barely enough tenacity to adhere to the inflamed surface, but the air in respiration is passed hurriedly over it, and it looses continually its water of solution, hence, is continually growing denser from this cause upon the outer surface; while an increased and increasing inflammation makes it more dense upon the inner surface also. And when the membrane once begins to form, this process is to go on in this manner until life is destroyed, or the disease arrested by medication; or in some rare and favorable cases, as before intimated, where fortunate chemical changes take place in the deposit. As for instance, when a still higher grade of inflammation causes pus to be thrown between the fibrinous deposit and the inflamed membrane, thus separating and detaching the cause of danger; or perhaps this same result may be obtained by the long contact of the false membrane with the mucous surface, acting like a foreign substance to induce pus secretion. Cures have undoubtedly followed pus secretion from this mode of action, when the inflamed surface was not too extensive, or did not extend to the minute divisions of the bronchia. But this condition should never be aimed at as a method of cure.

The more extensive the seat of inflammation, the more dangerous does the disease become; and especially when it extends downward into the sub-divisions of the bronchia and air cells. In which case the disease requires the most active and energetic treatment which the patient can bear.

And the same when the inflammation extends to the submucous or areolar tissue, whether the first of the inflammation is in the mucous membrane of the trachea, larynx, or fauces, or whether it commences in the areolar tissue, and extends to the mucous membrane, this complication is a dangerous one when not duly understood and appreciated, as is evidenced by the every day occurrence of death from diphtheria, which is nothing more than an inflammation of the areolar tissue beneath the mucous membrane of the parts above named, of an erysipletous character perhaps; whether it takes the erysipelatous character from the fact that the adjacent inflamed mucous membrane drains the blood of fibrin, or whether the inflammation is originally of the erysipelatous character, instead of the phlegmonous, is not my purpose to stop to inquire; but rather to make such suggestions as may, if possible, tend to lessen the mortality attending this complication of inflammation, as well as of uncomplicated croup. For I certainly feel warranted in saying, that the mortality in either is generally of a much larger ratio than should be found in this age of medical science; and its treatment should be based upon true pathological and therapeutic principles. When inflammation of areolar tissue about the throat occurs, especially if it be of the diffuse kind, it rapidly extends to the sheaths of large blood vessels and nerves of the part, and through these sheaths their contents must be more or less affected, and no physician need be told the importance of these parts to life, or the danger to the individual when they are subjected to active inflammation; or of the rapidity with which such inflammation must destroy life. Hence those sudden deaths which occur in diphtheria are what might be expected when we see important parts involved in a rapid inflammation. The rapidity of the extension of the inflammation shows it to be of diffuse character, while the diphtheric deposit upon the mucous membrane shows the grade of inflammation, as I have before attempted to show.

It is also true, that if the inflammation is not too active, and the resistant powers of the constitution are pretty good, that this inflammation may continue until ulceration of the mucous tissue and sub-cutaneous tissue occurs, and thus constitute what has been called malignant ulcerated throat; and in nothing differing from those laws of pathology which govern all cases of this kind of inflammatory action when located in parts so important. We could find the same grade of inflammatory action in the nose, in the rectum, and in the vagina, and feel no particular apprehension for the life of the patient, for the reasons that the same exudation upon the mucous membrane of the parts would not destroy life mechanically, or its extension in their own immediate neighborhood find organs so delicate and important to life as the par vagum.

This, then, is always in the onset an active inflammation of a rapid and dangerous character, both from the intensity of the disease, and the importance of the secretion, and especially when diphtheritic deposit blocks the trachea; or the functions of the par vagum are invaded by acute inflammation. In the latter case it too often happens that the pulse, respiration, and vital functions simulate debility, or rather the nervous functions and power of the lungs, heart, and stomach are depressed, while there is enough of vitality of the sympathetic nerves to keep up the local inflammation which goes on to the destruction of life, while the vital powers seem feeble and exhausted, and indeed are so for the want of the full force of their accustomed nervous functions. Hence, we may get fatal congestions of vital organs as a result of enervation, dependent on the causes above named. But it is active inflammation. And when such a liability occurs, we are to treat both the local inflammation, and whatever there may be of congestion.

This being an active inflammation, is to be cured by remedies, and not watched and guided through a certain succession of phenomena like small pox, measles, and scarlet fever. But it requires active and adequate treatment. A treatment that is sufficient to arrest inflammation; and any antiphlogistic treatment which falls short of this result operates to exhaust the patient, without arresting the disease, and absolutely shortens the period of the patient's existence; when a more energetic treatment would comparatively free the patient from danger in both croup and diphtheria.

Nor is a tonic course to be tolerated in either, and it could just as soon be tolerated in croup as in diphtheria. Neither should the doctrine of specific remedies, or empiricism, be justified in any one in this enlightened age, when a rational pathology and its therapeutic indications are attainable by all who are fit to practice medicine. Indeed I would pity the man who treats either of these diseases by giving a remedy because it is *good* in this disease; who has an idea that certain remedies thrown into the system, will course the circles of circulation, and follow the nervous expansions to their ultimate termination, searching for the offending particles of disease as a ferrit searches for rats in a hay stack, and having found the offending material, proceeds by some *hocos pocos* arrangement to give battle; be the particles of disease much, and of the remedy small, or *vice versa*, or with an idea that as the combat rages more remedy is to be thrown in from time to time, until it has searched and routed the whole disease from the system; my credulity is not large enough to endow any remedy found in the materia medica with intelligence and discretion beyond what a physician ought to have. Hence, I take the ground that the physician should know the disease in its different stages of structure and indications of cure.

And the first indication is to arrest the force of, and equalize the circulation. Second, restore normal secretion of the mucous membrane, and all secreting organs.

The older members of the profession can remember when it was considered necessary to bleed for all active inflammations, and that the amount of blood taken was of less importance than the impression made on the general system, and to get the full effect of bleeding as an antiphlogistic, it was necessary to place the patient upright, and take blood in a full stream until approaching syncope was felt; or in other words, suddenly abstract blood until the heart and muscular coat of the arteries failed to contract fast enough to keep that pressure upon the brain necessary to vitality; and this was to be repeated again in a few hours if the pulse became hard, and a complete reaction had taken place; and it was the extent of the impression made upon the brain and nervous functions—not the lessening of the quantity of the blood—that gave efficiency to the practice. It was the *enervating* effect that was sought by the remedy. With the march of improvement, since the resources of medication are better known, physicians do not find it necessary to bleed in active inflammations as formerly, and hence, even those who regard this as a highly inflammatory disease, have generally substituted some medication instead.

And although all, or nearly all, agree to lay aside the lancet in this condition of the mucous membrane; the profession are not agreed in the medication of this condition. Hence, the empiricism used in the treatment by those who would be aggrieved to be called *empirics*, and still they publish over their own signatures cures made by this and that remedy, leaving the full impression with the reader that the remedy knew more of the disease than the physician who prescribed it; and all that was necessary was to get a sufficient amount of the remedy into the system, and it would either like a chemical neutralizer destroy the disease, or the disease was invulnerable and not to be destroyed.

But if my premises are correct, we have a definite object to accomplish, and sufficient data and land-marks to know when they are accomplished.

The first then, may be the general enervation of the system to that degree necessary to arrest inflammation. And we all know that tartarized antimony possesses this power in an eminent degree; and to produce this effect of enervation it should be given in the manner indicated by the Italians, by the term "*tolerance*," not with a view to its emetic effect, for the reason, that when once you have got the stomach to reject medicine, you have lost all control of the disease by medication, if the case is a severe one; and so of cathartics. I have been called in consultation in bad cases of croup, and founded my prognosis entirely upon the fact of emesis or catharsis, having, or not having been induced, and can now recollect of no instance in which a judgment founded upon these premises was not sustained,—not that every case that is "puked" or "physicked" will die, because often either of these, or a less potent medication may arrest cases of mild croup in the incipient stages of the disease. In a bad case of croup, the antimony alone may not be sufficient to reduce the inflammation as speedily as may be desirable; in such cases we have an invaluable auxillary in the veratrum viride, a medicine which, when properly taken, operates directly to arrest the power of the nervous functions, and through this the force of the circulation. With these two remedies all inflammations of the air passages may be speedily subdued, if given before the period of congestions from the want of proper changes in the blood.

The antimony, then, is to be given in a dose just short of nausea, and may be repeated in fifteen minutes if the case is an urgent one, and after two or three doses are given, it can be borne in three or four times its ordinary dose if necessary, and without emesis, provided fluids are sparingly used. Given in this way it is a powerful sedative; it breaks down and prevents the formation of fibrine in the blood, and causes a thin aqueous secretion to be thrown from the inflamed vessels, instead of the fibrinous one which forms the false membrane; and when this membrane is formed, detaches it by being exuded between the mucous membrane and the deposit. Thus this medication operates for a three-fold purpose; to arrest the formation of fibrine; to arrest the inflammation, and to detach the membrane when formed. And it only needs the argument of experience and careful observation to be convinced of these facts. And from three to six hours are usually sufficient to accomplish these results. When the false membrane is once formed, there may be two ways of getting rid of it; the one above named, and in this way may also be included the slower process where the inflammation subsides by a slower process, and this state of secretion takes place more slowly; and where the false membrane becomes softened by incipient decomposition, and its solution, or partial solution, in the secretions of the more healthy membranes around it. And the second is where the inflammation passes to a higher grade, and *pus* is formed after the false membrane is deposited, and this pus being formed on the surface of the mucous membrane, and in those emunctories which have thrown out the fibrinous deposit, necessarily detaches the membrane. And it is not improbable that a long continued contact of the false membrane with the inflamed surface, operates like any other foreign substance to induce a pus formation beneath it. And hence *natures* operations may be even here restorative, where the amount of deposit is not too large, or the trachea is large enough to tolerate the secretion, and the inflammation does not extend too far toward the air cells.

Most of those cases where the membrane is detached after several days, are probably of this class of pus detachment. In a case of croup in a child two or three years old, we may not unfrequently find the pulse hard and frequent, to the number of one hundred and fifty even; in these cases there is no definite dose of veratrum to be given, but only the effect of the remedy is to govern the dose, and that effect should be to bring the pulse to be *soft*, and ten to twenty less in frequency than the healthy rate, and when this is done, with these remedies carefully given and watched, the inflammation speedily subsides.

Different preparations of mercury have long been used in this disease, some specifically, some empyrically, according to the physicians notions, or having no notions of the pathology of this disease. Judicious administration of the mercurials may assist to break down the fibrine of the blood, if needed, but they are less speedy in their operations than the remedies named for this purpose, and when any of the harsher preparations operate as *enervatives*, they do so by the *irritant* effect on the mucous membrane of the stomach, producing nausea; and not by any direct effect on the nervous structures and function as in the remedies named. Hence, although they, like a thousand other things, may cure croup, they are not a scientific legitimate prescription.

External applications are often used in croup with advantage, or disadvantage, according to the selections made. In croup, until the system is brought under the influence of remedies, the skin is dry, hot and harsh, and is in a pathological condition, and its removal is one of the steps toward arresting the inflammation. Perhaps nothing tends more quickly to induce the action sought than the "hydropathic warm bath," applied around the throat and upper part of the chest; to do which let some four or five thicknesses of common cotton cloth be wrung from very cold water so dry that it will not drip, spread smoothly over the skin above named, and cover quickly with cotton batting or wadding, sufficiently thick to keep the wet cloth warm; it speedily opens the pores, abstracts the heat, relieves the tension of the arteries, and is in fact a powerful antiphlogistic.

The snuff plaster, made of snuff and lard, has long been used in this disease, more as a popular remedy than one prescribed by physicians: perhaps when the skin is not too dry to absorb the strength of the tobacco, it has been of some use in enervating the system generally, and thus operate to some extent as an arterial sedative. It cannot be needed where the veratrum is used. Mustard, pepper, salt, or any other remedy which operates to stimulate the parts, does harm by constringing the already too tense vessels of the part; so of blisters, early in the disease. Warm applications about the throat are of doubtful value; they may, it is true, relax the skin, but they invite more blood to the part without any corresponding benefit.

The instances in croup which demand tracheotomy are of very rare occurrence; it can only be used where the obstruction is in the larynx, or when the loose diphtheritic deposit in the trachea is of such size or form at to prevent its being passed through the larynx.

And I can conceive of no condition which would justify me in opening the trachea for the purpose of medicated injections, when as good results can be more speedily obtained by general medication. And I am of the same opinion in the laryngial variety of croup.

We have often an inflammation of the fauces, pharynx, larynx, and subjacent areolar tissue, called diphtheria, in which we have this same deposit of the results of inflammation upon the mucous membrane, while the inflammation is extended to the parts beneath. I apprehend there is often an error in making up the mind of the true pathology of this disease, for so long as the mucous membrane is in such a state of inflammation as to throw out the diphtheritic deposit, it is most certainly sthenic, or rather, inflammatory action, as in no other condition of the mucous membrane is this deposit thrown out; and I find it very *hard* to understand when the mucous membrane is in this particular and definable state of inflammation, how the contiguous areolar tissue can be suffering from asthenic action, or disease of debility; or in other words, how parts not one-fiftieth of an inch distant can be suffering, the one from active inflammation, and the other from the want of even normal tone. Nor do I believe that such a condition ever exists. But that the sub-mucous tissue is affected with inflammatory action the same as the mucous membrane, and that the same treatment which is efficient to remove the inflammation of the one, will remove it in the other also. And so long as there is any tendency to the diphtheric deposit, there should be no doubt as to the sthenic or asthenic character of the disease. And it not unfrequently happens, that in a case which has a hard *small* pulse, one so small as even to make many doubt the sthenic character of the disease, that under the influence of the remedies above indicated the pulse becomes full, slow, and soft, with all of the consequent accompanying phenomena; while under a tonic course the pulse becomes harder and smaller as inflammation advances, until congestion of vital organs ensues.

In croup many depend upon the stimulant expectorants in the early stages; as upon the compound known through the country, and retailed from almost every druggist under the name of "Hive Syrup," a thing, which but for the emetic tartar it contains would be so obviously pernicious as to be universally discarded.

I would only add that the squills and seneka, and all like remedies, have no place in curing active inflammations of mucous membranes.

We have certain epidemic conditions of the circulating fluids which sometimes terminate in erysipelatous action; this erysipelas may show itself in the cellular tissue of the throat, or any other part, as of a leg or an arm; and is a malignant disease in which the vital powers of the part affected, rapidly, sometimes in less than twelve hours, pass into a state of gangrene and decomposition, with but a very short period of pain, and with but very slight general excitement. When in the throat it may be called malignant sore throat, and is quite a different thing from diphtheria.

In diphtheria we have an active antiphlogistic in the use of the solution of nitrate of silver, applied to the inflamed parts by means of the probang. The solution should be at least twenty grains to the ounce; and after the skin is moist by the remedies before named, a few applications completely relieve the soreness and remaining inflammation by its power of lessening the nervous sensibility of the inflamed part, and contracting the congested capilaries to their normal condition.

The duration of diphtheric inflammation depends upon the character and efficiency of the treatment. If treated from the first with tonics and specifics, the duration may be for days and weeks. If treated as above indicated, a few hours suffices to arrest the inflammation, and a day or two more for its cure.

And with regard to debility—the bug-bear which has destroyed its thousands—there is none of it, so long as the peculiar diphtheric deposit is manifested, whether in the fauces, larynx, or trachea; and this deposit alone may be taken as evidence of active inflammation.

